# The NPC Families Mediate BmNPV Entry

**DOI:** 10.1128/spectrum.00917-22

**Published:** 2022-07-05

**Authors:** Youpeng Fan, Jialing Bao, Xiaoyu Fu, Pengfei Wu, Jingya Chen, Yan Huang, Junhong Wei, Guoqing Pan, Chunfeng Li, Zeyang Zhou

**Affiliations:** a State Key Laboratory of Silkworm Genome Biology, Southwest University, Chongqing, China; b Chongqing Key Laboratory of Microsporidia Infection and Prevention, Southwest University, Chongqing, China; c College of Sericulture, Textile and Biomass Sciences, Southwest University, Chongqing, China; d College of Life Sciences, Chongqing Normal University, Chongqing, China; University of Arizona

**Keywords:** *Bombyx mori*, BmNPV, NPC1, NPC2, cholesterol trafficking pathway, enveloped virus

## Abstract

Baculovirus is a powerful tool for biological control in agriculture and foreign gene expression and delivery in insect and mammalian cells. Baculovirus enters host cells by multiple endocytic pathways; however, the current understanding of the Bombyx mori nucleopolyhedrovirus (BmNPV) entry mechanism remains limited. Previous studies have identified NPC1 and NPC2 as important host factors for viral infection in insect cells, although their exact role in viral infection has not yet been determined. In this study, we demonstrate that the BmNPC1 protein is an important intracellular factor for BmNPV escape from the endosomal compartment, and the expression of BmNPC1 in Sf9 cells confers the virus the ability to enter into the nucleus of Sf9 cells. Additionally, the second luminal domain of BmNPC1 (BmNPC1-C) binds to the viral glycoprotein gp64, and preincubation of BmNPV with purified BmNPC1-C inhibits virus infection. Furthermore, knockout of the BmNPC2 protein results in reduced efficiency of viral fusion with the endosomal membrane, and BmNPC2 protein interacts directly with both viral envelope glycoprotein gp64 and the host BmNPC1 protein. BmNPC2 was found to be incorporated into progeny viral particles. Taken together, our results suggest that NPC2 protein incorporated into viral particles may facilitate viral infection through promoting the interaction of BmNPV and NPC1 in the endosome, thus enhancing viral fusion and escape from endosomes. These results, combined with those from previous studies, support that BmNPV hijacks two important cholesterol receptor members (NPC1 and NCP2) in the cholesterol intracellular transport pathway for viral entry into host cells.

**IMPORTANCE** Baculovirus is an important biological factor for controlling insect populations and represents a powerful biological tool for gene delivery and expression. However, the host receptor of baculovirus is still unknown. In this study, we demonstrate that BmNPC1 protein is an important intracellular factor for BmNPV escape from the endosomal compartment, and the expression of BmNPC1 confers the ability of virus to enter into the host cell nucleus in nonpermissive Sf9 cells. BmNPC2 can bind to the virus and promote progeny virion infection through the NPC1-NPC2 endosome cholesterol transport pathway. We believe that our study on the BmNPV entry mechanism will further facilitate the application of baculovirus systems in eukaryotic gene delivery. Not only can the cholesterol transport pathway NPC1 protein be used by a variety of enveloped viruses, but the NPC2 protein can also be used by viruses to infect host cells. This will provide new insights into the study of enveloped virus infection mechanisms.

## INTRODUCTION

Enveloped viruses infect host cells by binding to the cell surface and delivering their genetic material into the cytoplasm. The fusion of the viral envelope with the host membrane is essential for virus to bypass the cellular membrane and release nucleocapsids into the cytoplasm (1). Baculoviruses are rod shaped, 230 to 385 nm in length, and 40 to 60 nm in diameter that infect insects of the orders *Lepidoptera*, *Diptera*, and *Hymenoptera* (2). Autographa californica multiple nucleopolyhedrovirus (AcMNPV) and Bombyx mori nucleopolyhedrovirus (BmNPV) are group I alphabaculoviruses that have acquired the gp64 protein to mediate membrane fusion during evolution (3). Although the BmNPV genome shares over 90% identity with that of AcMNPV, AcMNPV tends to have wider host ranges (4). Baculovirus enters host cells by clathrin-mediated endocytosis and macropinocytosis (5, 6). The fusion between the viral membrane and host endosomal membrane is mediated by the viral gp64 protein and triggered by the low pH in the endosomal lumen (7). The exact identities of host receptors for baculovirus remain elusive; heparin sulfate (8), phospholipids (9), and Bombyx mori receptor expression-enhancing protein (BmREEPa) (10) all have been suggested to be involved in BmNPV binding or attachment.

Our previous study suggested that Niemann-Pick C1 (NPC1) is an essential host factor for baculovirus entry and infection in cells of the *B. mori* cell line BmE (11). NPC1, a key member of the cholesterol receptor family, is a large polytopic membrane protein that resides in the cytomembrane and late endosomes in BmE cells and is involved in lysosomal cholesterol transport to the endoplasmic reticulum and other cellular sites (12). NPC2, another member of the cholesterol receptor family, takes up cholesterol from the intraluminal vesicles in late endosome/lysosome (LE/Ly) compartments and then transports cholesterol to NPC1 for downstream cross-membrane transport (13). These two proteins work cooperatively to regulate cholesterol egress from LE/Ly (14–16), and local cholesterol accumulation could result from a loss of function in either protein (17). NPC1 protein contains three luminal domains, including the amino-terminal domain (NTD), the C domain (also known as the luminal domain; MLD), and the I domain (also known as the CTD), and 13 transmembrane channels (13, 18). Among these, the NTD has been suggested to be able to bind sterol analogs or cholesterol; domain C can interact with NPC2 (19, 20). The NPC2 crystal structure suggests that the hydrocarbon tail of cholesterol inserts deeply into the binding pocket of NPC2, exposing cholesterol to only the hydroxyl group in the lysosomal lumen (21). Cholesterol is solubilized for handover to NPC1 (NTD), which binds cholesterol from the hydroxyl group (15). Furthermore, systematic structural characterizations have revealed the molecular basis for the low-pH-dependent cholesterol delivery from NPC2 to NPC1 transmembrane domain (13). BmNPC1 encoding a protein of 1,334 amino acids shares 43% sequence similarity with hNPC1, indicating that this gene is a potential homolog of a vertebrate NPC1 family member and contains 13 transmembrane-spanning helices and 3 large luminal loops, namely, domain A (residues 1 to 270), domain C (residues 408 to 645), and domain I (residues 881 to 1136), like hNPC1.

It has been revealed that NPC1 plays a role in the entry of several viruses. Ebola virus is internalized and transported to the LE/Ly, in which the glycoprotein is proteolytically processed to specifically bind to NPC1, promoting viral escape from the endosomal compartment (22–24). Human immunodeficiency virus type 1 and hepatitis A virus also depend on the NPC1-mediated intracellular cholesterol trafficking pathway for productive viral infection (25, 26). In our previous study, we demonstrated that baculovirus entry and infection in BmE cells rely on the host factor NPC1 and that gp64 can interact with NPC1 directly (11). Additionally, it has been reported that BmNPV infection can be enhanced by Bombyx mori promoting protein, a family member of Niemann-Pick C2 (NPC2), in silkworm BoMo cells (27, 28). The addition of BmNPC2 to culture media at a concentration of 1 μg/mL resulted in a 1,000- to 10,000-fold rise in BmNPV production (27). However, regarding how an NCP2 family member contributes to BmNPV infection, this remains elusive. The BmNPC2 protein was identified as a single polypeptide of 15.2 kDa. In silkworm larvae, NPC2 is possibly synthesized in the fat body and then secreted into the hemolymph. Injection of NPC2 into living silkworms increased triglyceride levels in the fat body (29).

In this study, we demonstrate that NPC1 is an important host factor contributing to viral fusion and escape from the endosomal compartments, and the depletion of NPC1 inhibits BmNPV entry into the nucleus. When BmNPC1 was expressed in a nonpermissive Sf9 cell line, BmNPV acquired the ability to enter the nucleus. After incubating the virus with purified NPC1-C domain protein, the virus infectivity decreased by 80%, indicating that the infection of baculovirus depends on the NPC1-C region interaction. Knockout of NPC2 reduced the fusion efficiency of the viral membrane and host endosomal membrane, and the expression of NPC2 protein in the NPC1 knockout cell line did not promote virus infection. Altogether, our results indicate that NPC1 is an essential intracellular factor for BmNPV infection and promotes a late step in viral entry by binding to the viral glycoprotein gp64, and NPC2 protein facilitates viral infection of host cells through the NPC2-NPC1 endosomal lysosomal cholesterol transport pathway.

## RESULTS

### Inefficient virus-endosome fusion limits BmNPV entry into NPC1-null cells.

**FIG 1:**
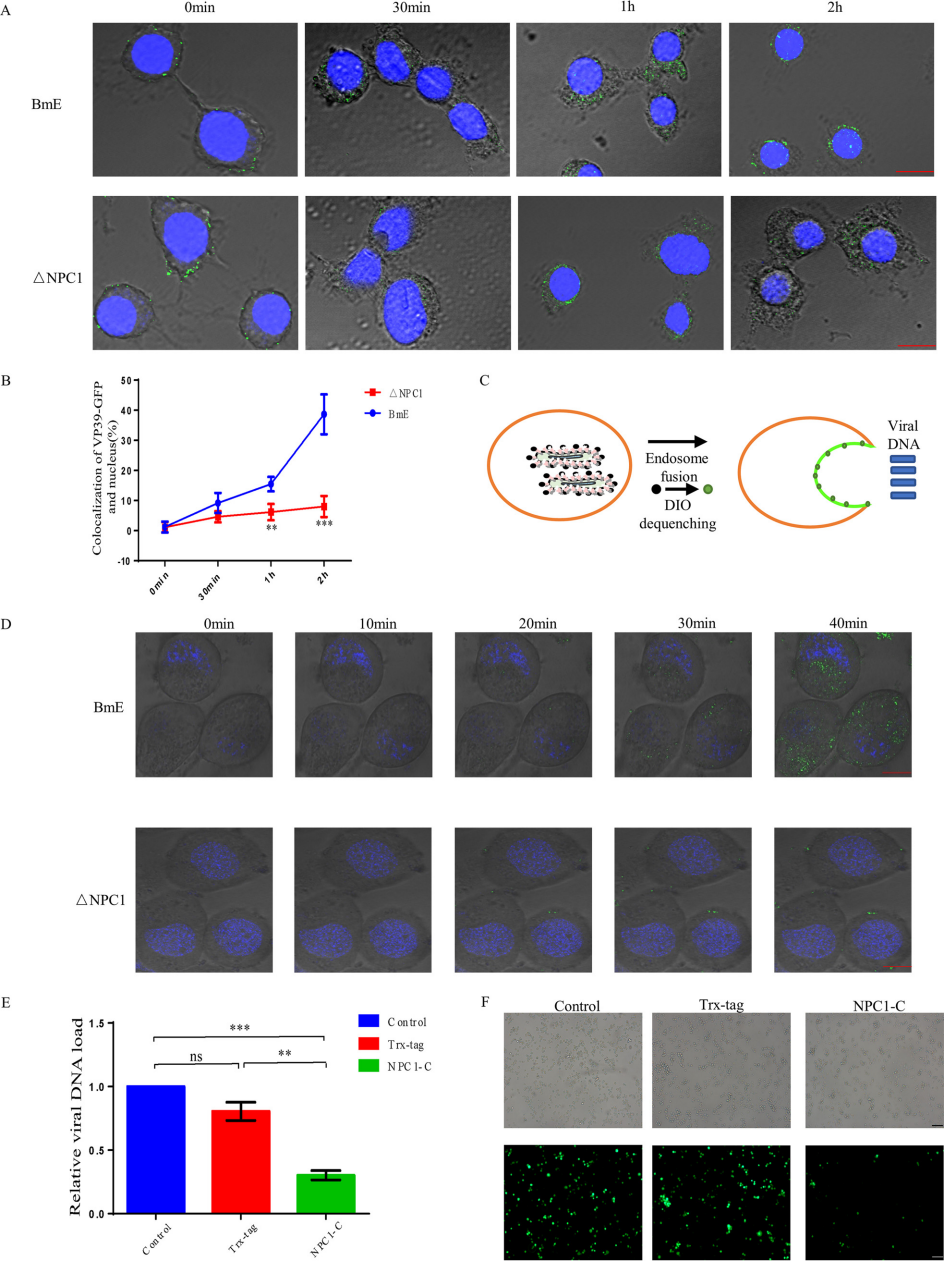
Inefficient virus-endosome fusion limits BmNPV entry into NPC1-null cells. (A) Localization of BmNPV in BmE cells and NPC1-null cells. BmE cells were permeabilized (30 min, 1 h, 2 h) and unpermeabilized (0 min). Green fluorescence was observed in the samples treated with anti-EGFP antibodies against BmNPV VP39-EGFP. The nucleus was stained by DAPI. (B) Five fields were randomly selected to use ImageJ software to analyze the colocalization rate of the virus and nucleus in each cell. Statistical analysis was conducted with paired two-tailed *t* tests. *, *P < *0.05; **, *P < *0.01; ***, *P < *0.001. Data are means ± SD (*n* = 3). (C) Illustration of the experiment performed in panel D. (D) Confocal imaging showed the delivery of DiO-labeled virus (green). DiO-labeled BmNPV (MOI, 80) was bound to BmE and BmNPC1-null cells for 1 h at 4°C. Then, cells were shifted to 28°C for the indicated times. In the end, cells were stained with Hoechst 33342 (blue), and DiO fluorescence was quantified by confocal microscopy. (E) BmNPV was incubated with the purified NPC1-C protein or Trx-tag protein-infected cells at an MOI of 1 for 72 h. Then, the relative viral DNA load was tested at 72 h p.i. Statistical analysis was conducted with a one-way ANOVA followed by Dunn’s multiple comparison test. *, *P < *0.05; **, *P < *0.01; ***, *P < *0.001. Data are means ± SD (*n* = 3). (F) Viral infection was indicated by positive EGFP expression in BmE cells. Bar, 200 μm.

In our previous study, we demonstrated that when BmNPC1 is absent, baculoviruses are blocked at the early stage of viral infection. Here, we examined at which step viral infection is affected by the absence of BmNPC1. For this purpose, BmE cells and NPC1-null cells were inoculated with virus VP39 labeled with enhanced green fluorescent protein (VP39-EGFP) at a multiplicity of infection (MOI) of 80 at 4°C for 1h, then shifted to 28°C for 2 h post-virus inoculation before being imaged by confocal microscopy. The localization of VP39-EGFP in the nucleus was quantified. As shown in Fig. 1A and B, in control cells, the localization of VP39-EGFP in the nucleus steadily increased by 2 h postinoculation, whereas in NPC1-null cells, VP39-EGFP signals largely remained in the cytosol and failed to reach the nucleus with the progression of incubation. We conclude that knocking out BmNPC1 in BmE cells can inhibit viral entry into the nucleus. Certain viruses, including baculovirus, flaviviruses, such as dengue virus and yellow fever virus, and Ebola virus, require transport from early endosomes to endocytic carrier vesicles or late endosomes for efficient endosomal escape. To determine whether BmNPC1 affects the efficiency of virus fusion with endosomal membrane, we tracked viral fusion with DiO-labeled BmNPV particles. DiO is a lipophilic, self-quenching dye and has been widely used for studying enveloped virus fusion kinetics. Fusion of the labeled virus with a host membrane causes dequenching of DiO, which can be observed by confocal microscopy (Fig. 1C). BmE cells or BmNPC1 knockout cells were incubated with BmNPV at an MOI of 80 at 4°C for 1 h, followed by inducing virus internalization for 40 min by shifting the temperature to 28°C. After 40 min of internalization, there were substantial increases in green fluorescent signal indicative of efficient viral fusion in the endosomes in control cells (Fig. 1D); in contrast, in BmNPC1 knockout cells, the fluorescent signals were not obvious. Taken together, our results suggest that BmNPC1 plays an essential role in viral fusion. It has been shown previously that the NPC1-C domain is necessary for gp64-NPC1 binding and viral entry mediated by baculovirus gp64 (11). We next investigated whether the BmNPC1-C domain could bind to gp64 and block virus infection. We incubated the purified BmNPC1-C proteins with BmNPV at 4°C for 8 h before adding the mixture to the cells and found that incubated virus with BmNPC1-C protein reduced virus infectivity (Fig. 1E and F). These findings unequivocally separate NPC1-C’s functions in lysosomal cholesterol transport and baculovirus entry.

### Expression of BmNPC1 confers BmNPV with the ability to enter nuclei of a nonpermissive Sf9 cell line.

**FIG 2:**
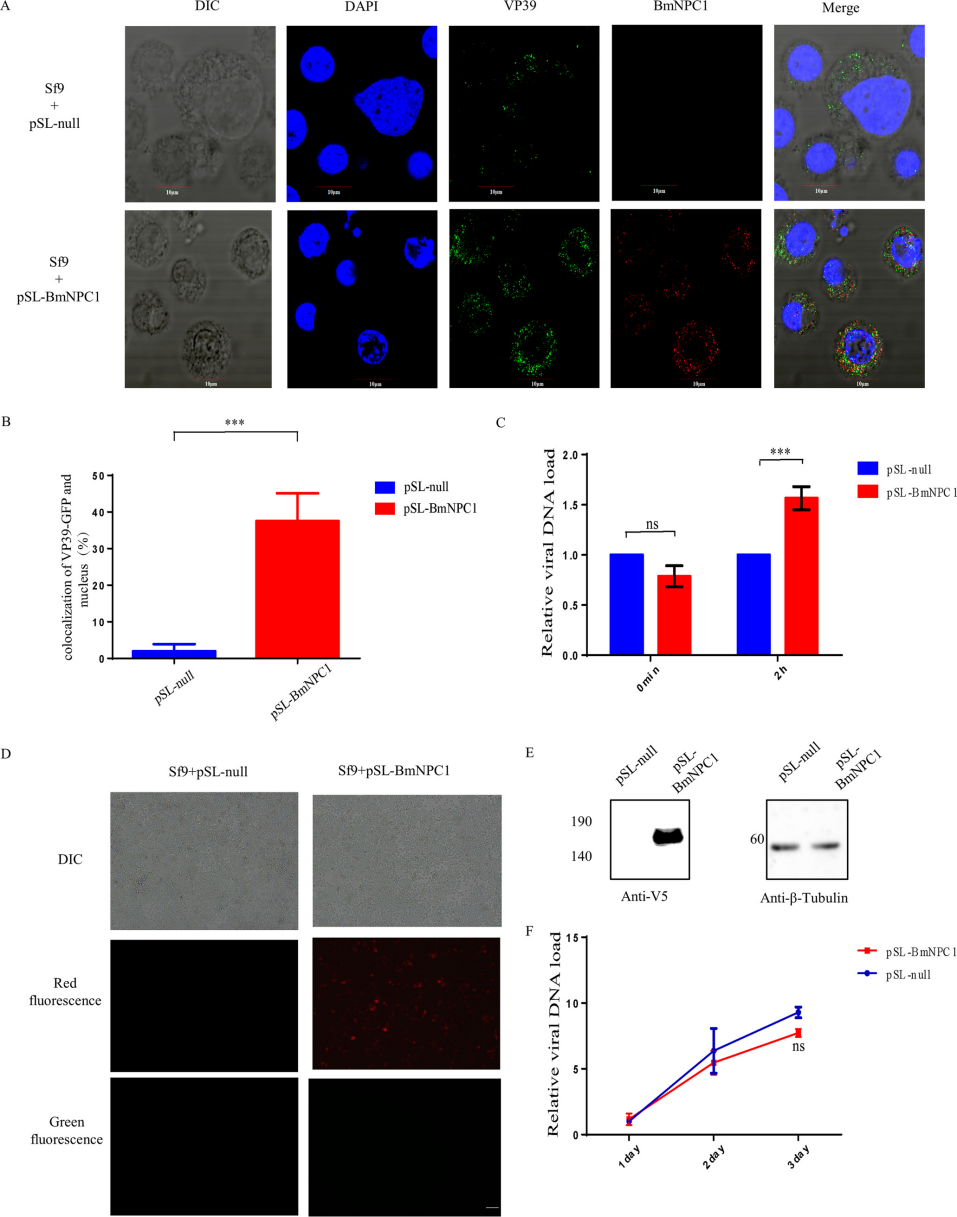
Expression of BmNPC1 confers BmNPV with the ability to enter the nuclei of a nonpermissive Sf9 cell line. (A) Sf9 cells were transfected with pSL1180-IE2-BmNPC1-mCherry(pSL-BmNPC1-mCherry) or a pSL1180-null(pSL-null) vector, then after 3 days infected with VP39-EGFP virus for 2 h, following the protocols for the live-cell imaging. (B) We used ImageJ software to analyze the colocalization rate of the virus and nucleus in each cell. Statistical analysis was conducted with paired two-tailed *t* tests. ***, *P < *0.001. Data are means ± SD (*n* = 3). (C) BmNPV was bound to Sf9 cells expressing BmNPC1 at an MOI of 80 for 1 h at 4°C, followed by inducing internalization for 2 h. The cells were washed and lysed. Total DNA was extracted from infected cells, and the BmNPV viral DNA level was determined by real-time quantitative PCR. Sf9 *gadph* was used as the endogenous control. Statistical analysis was conducted with paired two-tailed *t* tests. ***, *P < *0.001. Data are means ± SD (*n* = 3). (D) Fluorescence microscopy of BmNPV (green fluorescence)-infected transfected pSL-null Sf9 cells and pSL-BmNPC1-mCherry (red fluorescence)-expressing Sf9 cells at 72 h p.i. Expressed BmNPC1-mCherry Sf9 cells were infected with BmNPV at an MOI of 3, and we detected BmNPV infection after 3 days. (E) BmNPC1-mCherry and *S. frugiperda* beta-tubulin in cell lysates were detected by Western blotting using specific antibodies. (F) The relative viral DNA load was determined by qPCR analysis using *gp41* primers; the viral DNA load in Sf9 cells at the indicated times at 24 h were set as 1. Statistical analysis was conducted with paired two-tailed *t* tests. ns, not significant.

BmNPV exhibits distinct permissibility in different types of insect cells, with the Bombyx mori cell line being broadly susceptible to viral infection and Sf9 cell line derived from Spodoptera frugiperda being nonpermissive. Based on our recent finding of BmNPC1 being a critical host factor for baculovirus fusion in the endosome, we next examined whether the expression of exogenous BmNPC1 in Sf9 cells can grant permissibility to BmNPV infection. We transfected pSL-BmNPC1-mCherry vector in Sf9 cells for 2 days, then inoculated the cells with VP39-EGFP at an MOI of 80 at 4°C for 1 h, followed by inducing internalization for 2 h. As shown in Fig. 2A, BmNPV viruses represented by VP39-EGFP signals were predominantly located in the cytoplasm at 2 h postinternalization in control Sf9 cells, whereas in Sf9 cells expressing BmNPC1, a fraction of viruses were successfully translocated in the nucleus, indicating the expression of BmNPC1 can promote virus escape from LE/Ly (Fig. 2B). To determine whether BmNPC1 modulates attachment and entry of budding virus (BV) to cells, control or BmNPC1-expressing Sf9 cells were precooled at 4°C for 30 min, then inoculated with BmNPV (MOI, 80) at 4°C for 60 min, and we quantified cell-bound virus by quantitative PCR. Then, we shifted a subset of cells to 28°C for 2 h and assessed viral entry by quantification of viral gene copy numbers. Cells were collected at 0 h and 2 h and washed with cold phosphate-buffered saline (PBS) 3 times to remove the unbound viruses, and the cell-bound virus or internalized viral load was determined by quantitative PCR (qPCR) (Fig. 2C). While BmNPC1 expression still enhanced viral entry under the temperature shift conditions, there was no significant difference in the number of viral genomes bound at 4°C to BmNPC1-expressing cells compared to control cells. Next, we assessed whether the translocation of BmNPV viruses into the nucleus induced by the exogenous expression of BmNPC1 could lead to virus replication in Sf9 cells (Fig. 2D). First, we used an anti-V5 antibody to detect the expression of BmNPC1(Fig. 2E). Then, we infected BmNPC1-expressing Sf9 cells with BmNPV at an MOI of 3 for 72 h and quantified viral gene copy numbers by qPCR (Fig. 2F). We found that the relative viral gene copy numbers were comparable in wild-type and BmNPC1-expressing Sf9 cells, suggesting additional barriers in viral infection at a post-nuclear translocation step in Sf9 cells and that overexpression of BmNPC1 alone is not sufficient for productive viral infection in this cell type.

### NPC2 enhances early steps of the viral life cycle.

**FIG 3:**
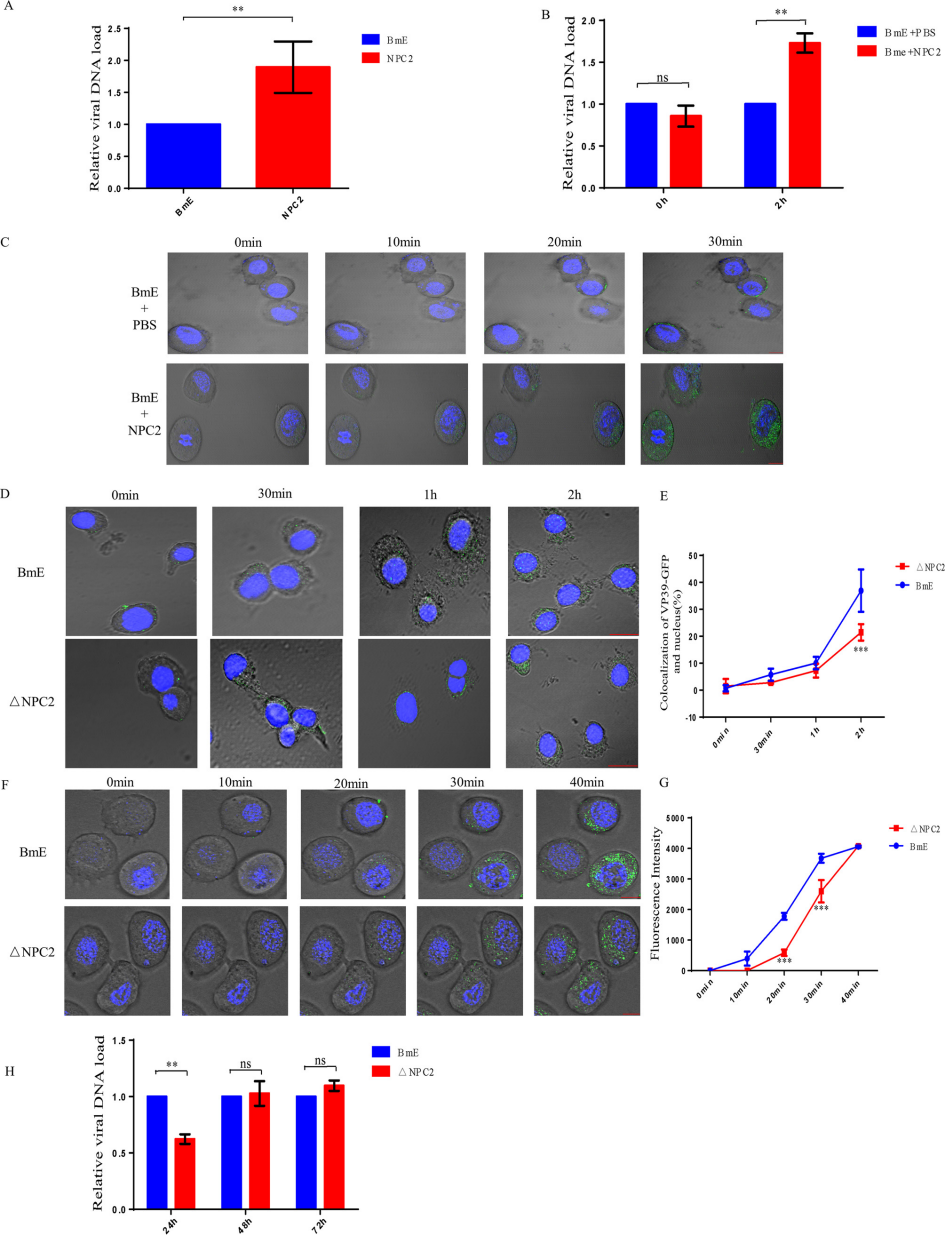
NPC2 enhances the early stages of the viral life cycle. (A) BmNPV multiplication was measured in BmNPC2-treated cells (10 μg) and BmE cells infected with BmNPV-EGFP at 72 h p.i by qPCR. Statistical analysis was conducted with paired two-tailed *t* tests. **, *P < *0.01. Data are means ± SD (*n* = 3). (B) The relative viral DNA load in NPC2-treated cells (10 μg) and wild-type cells at 0 h p.i. and 2 h p.i. Precooled NPC2-treated cells and wild-type cells were inoculated with BmNPV-EGFP virus, and the relative viral DNA loads were determined at 0 h p.i. and 2 h p.i. The viral DNA load in wild-type cells at the indicated times was set as 1. Statistical analysis was conducted with paired two-tailed *t* tests. *, *P < *0.05; **, *P < *0.01. Data are means ± SD (*n* = 3). (C) BmE cells and NPC2-treated cells (10 μg) bound DiO-labeled BmNPV (MOI, 80) for 1 h at 4°C. The cells were shifted to 28°C for the indicated times. The cells were stained with Hoechst 33342 (blue), and DiO fluorescence was quantified by confocal microscopy. (D) Localization of BmNPV in BmE cells and NPC2-null cells. Cells were permeabilized (30 min, 1 h, 2 h) or unpermeabilized (0 min). Green fluorescence was observed in the samples treated with anti-EGFP antibodies against BmNPV VP39-EGFP. The nucleus was stained with DAPI. (E) We used ImageJ software to analyze the colocalization rate of the virus and nucleus in each cell. Statistical analysis was conducted with paired two-tailed *t* tests. ***, *P < *0.001. Data are means ± SD (*n* = 3). (F) DiO-labeled BmNPV (MOI, 80) were bound to BmE and BmNPC2-null cells for 1 h at 4°C. The cells were shifted to 28°C for the indicated times. The cells were fixed with Hochest 33342 (blue), and DiO fluorescence was detected by confocal microscopy. (G) We used ImageJ software to analyze the fluorescence intensity. Statistical analysis was conducted with paired two-tailed *t* tests. *, *P* < 0.05; **, *P* < 0.01; ***, *P < *0.001. Data are means ± SD (*n* = 3). (H) Wild-type BmE cells and BmNPC2-null BmE cells (ΔNPC2) were inoculated with BmNPV, and cell samples were collected at the indicated times. BmNPV viral load was measured by qPCR analysis using gp41 DNA primers, and relative copy numbers were calculated using *B. mori gapdh* DNA as the internal control (MOI, 0.3), and the fold change of viral DNA load was determined at 24 h, 48 h, and 72 h postinfection. The viral DNA loads in wild-type cells at the indicated times were set as 1. Statistical analysis was conducted with paired two-tailed *t* tests. ns, not significant; *, *P < *0.05; **, *P < *0.01. Data are means ± SD (*n* = 3).

Previous studies showed that the addition of BmNPC2 in the culture medium enhances BmNPV infection in BoMo cells (27). Therefore, we investigated the mechanism of NPC2 protein to promote virus infection. For this purpose, BmE cells and NPC2-treated cells were infected with BmNPV (MOI, 0.5) for 72 h; we then quantified viral gene copy numbers by quantitative PCR and found that BmNPC2 promotes virus infection (Fig. 3A). The BmE cells and NPC2-treated cells were infected with BmNPV (MOI, 80) at 4°C for 1 h and then we quantified viral gene copy numbers by qPCR. Next, we shifted a subset of cells to 28°C for 2 h and assessed viral entry by quantifying viral gene copy numbers. We found that added NPC2 protein did not affect BmNPV binding but it did affect virus entry (Fig. 3B). To determine whether NPC2 affects the entry efficiency of virus endocytosed into cells, we tagged BmNPV with DiO, as previously described. We found differences in the number of fluorescent spots at time points during entry. On average, control cells had fewer endosome fusion signals at each time point compared to NPC2-treated cells (Fig. 3C). In order to dissect at which step or steps of the viral replication cycle BmNPC2 contributes to viral infection, we first generated a BmNPC2-null BmE cell line by CRISPR-Cas9 genome engineering, and the absence of its expression was confirmed by Western blotting (see Fig. S1C in the supplemental material). The resulting cholesterol accumulation in the cytoplasm was also verified by filipin staining (see Fig. S1B). In addition, knockout of NPC2 did not affect cell viability (see Fig. S1D). To examine whether the depletion of BmNPC2 affects BmNPV binding, BmNPC2-null cells and the control wild-type BmE cells were inoculated with VP39-EGFP viruses at an MOI of 80 for 1 h at 4°C, and we found that viral signals at the cell surface were comparable in these two types of cells, indicating that BmNPC2 has no significant effect on virus binding. Next, we induced viral internalization at 28°C. As shown in Fig. 3D and E, unlike the efficient viral translocation into the nucleus seen in control cells, the colocalization of VP39-EGFP with 4′,6-diamidio-2-phenylindole (DAPI) signal was moderately reduced in BmNPC2 null cells, suggesting that BmNPC2 plays a role in the early steps of viral infection. To further pinpoint the exact steps in which BmNPC2 plays a role in the early steps of viral infection, we used DiO-labeled BmNPV to observe viral fusion efficiency in real time and ImageJ software to analyze the fluorescence intensity. The results showed that the knockout of BmNPC2 affects the fusion efficiency of the viral envelope and endosome membranes, compared with knockout NPC2 cells, the signal for fusion of the viral envelope and cell membrane can be detected earlier in BmE cells (Fig. 3F and G). Together, these experiments show that NPC2 enhances an early stage in viral infection prior to replication. To determine whether knockout of NPC2 affects later stages of the viral life style, BmNPC2-null cells and the control wild-type BmE cells were infected with BmNPV (MOI, 0.3), and qPCR analysis showed that the viral gene copy numbers in BmNPC2-null cells were reduced after 24 h compared with controls, but no difference was observed at 48 or 72 h postinfection (Fig. 3H). Interestingly, our data indicated that after the knockout of NPC2, the efficiency of virus fusion decreased. However, after knockout of NPC1, the virus fusion efficiency was more inhibited. This suggests that the BmNPC1 protein is more important for virus fusion.

### BmNPC2 interacts with BmNPV gp64 and BmNPC1 protein.

**FIG 4:**
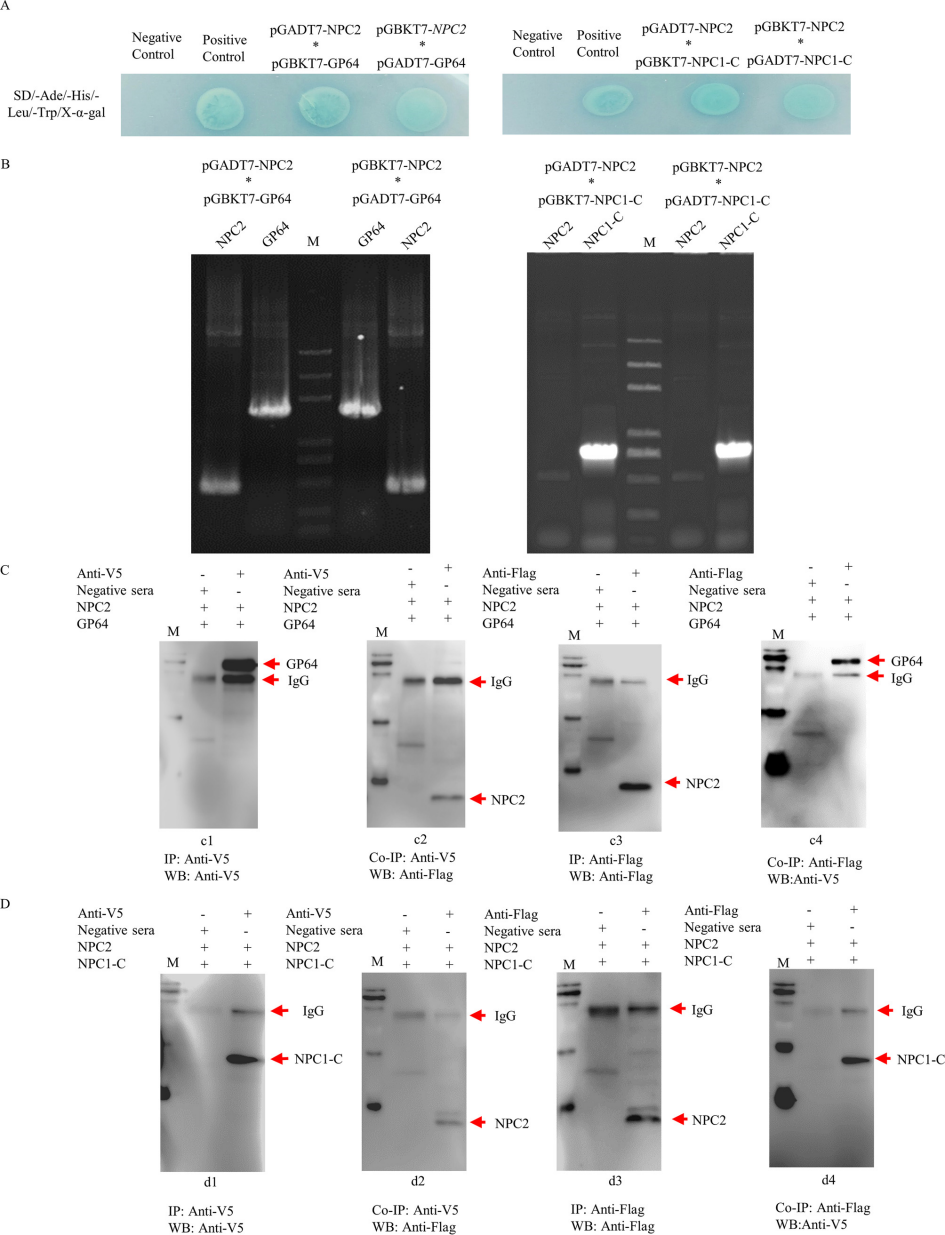
BmNPC2 interacts with BmNPV gp64 and BmNPC1 protein. (A and B) Yeast two-hybrid assay for determining the interaction of BmNPC2 and BmNPC1-C with gp64. pGADT7-BmNPC2 (prey)/pGBKT7-gp64 (bait), pGADT7-gp64 (prey)/pGBKT7-BmNPC2 (bait), pGADT7-BmNPC1-C (prey)/pGBKT7-NPC2 (bait), and pGADT7-NPC2 (prey)/pGBKT7-BmNPC1-C (bait) constructs were transformed into competent yeast cells. A number of independent blue colonies, including the pGADT7-BmNPC2 (prey)/pGBKT7-gp64 (bait), pGADT7-gp64 (prey)/pGBKT7-BmNPC2 (bait), pGADT7-BmNPC1-C (prey)/pGBKT7-NPC2 (bait), and pGADT7-NPC2 (prey)/pGBKT7-BmNPC1-C (bait) constructs grew on SD plates lacking Leu, Trp, His, and Ade but containing X-α-Gal. AH109[pGBKT7-53]/Y187[pGADT7-T] was used as a positive control, and AH109[pGBKT7-53]/Y187[pGKDT7-lam] was used as a negative control. Plasmids were extracted from transformed blue colonies and PCR was used to analyze gene expression. (C and D) BmNPC2 interacted with BmNPC1-C and BmNPV gp64 as detected by coimmunoprecipitation (co-IP). Subpanels c1, c3, d1, and d3 show the interaction between antibodies and expressed proteins as demonstrated by immunoprecipitation (IP). Anti-V5 or Anti-Flag monoclonal antibody was used for IP and Western blotting (WB). c1, gp64 incubated with BmNPC2; c3, BmNPC2 incubated with gp64; d1, BmNPC1-C incubated with BmNPC2; d3, BmNPC2 incubated with BmNPC1-C; lane M, EasySee Western blotting marker. Subpanels c2, c4, d2, and d4 show that BmNPC2 interacted with BmNPC1-C and BmNPV gp64 as detected by co-IP. For subpanels c2 and c4, anti-V5 or anti-Flag monoclonal antibody was used for IP with gp64 or BmNPC2. c2, GP64 incubated with BmNPC2; c4, BmNPC2 incubated with GP64. The anti-V5 antibody was used for co-IP with gp64; the anti-Flag antibody was used for co-IP with BmNPC2. For subpanels d2 and d4, anti-V5 or anti-Flag monoclonal antibody was used for IP with NPC1-C or BmNPC2. d2, BmNPC1-C incubated with BmNPC2; d4, BmNPC2 incubated with BmNPC1-C. The anti-V5 antibody was used for co-IP with BmNPC1-C, and the anti-Flag antibody was used for co-IP with BmNPC2.

Previous studies have shown that BmNPC1 can interact with baculovirus glycoprotein gp64, the primary glycoprotein responsible for receptor binding and fusion. To investigate whether BmNPC2 contributes to BmNPV infection through interacting with gp64 during viral entry, a yeast two-hybrid system (Y2H) was employed. Full-length NPC2 was cloned into the pGBKT7 vector as bait, and BmNPC1-C and gp64 were used as the prey. Y187[pGADKT7-NPC1-C]/AH109[pGBDT7-NPC2] and Y187[pGADKT7-gp64]/AH109[pGBDKT7-NPC2] yeast cells were constructed. The constructs grew on synthetic dropout medium (SD) plates lacking Leu, Trp, His, and Ade and containing 5-bromo-4-chloro-3-indoxyl-α-d-galactopyranoside (X-α-Gal) and turned blue after hydrolyzing X-α-Gal (Fig. 4A). The clones were screened, and plasmids were extracted from the transformed blue colonies for PCR. The PCR results showed that the positive blue colonies contained NPC2/NPC1-C and NPC2/gp64 genes (Fig. 4B). A yeast two-hybrid reverse experiment produced the same result. These Y2H screens revealed that BmNPC2 interacts with gp64. Collectively, these results demonstrate that NPC2 is sufficient for gp64 or NPC1-C binding. Together, these data suggest that NPC2 interacts with gp64 and promotes BmNPV infection in BmE cells. To further confirm these interactions, coimmunoprecipitation was performed with *in vitro*-expressed proteins. gp64 and NPC1-C-attached protein G-agarose beads through mouse anti-V5 antibody were incubated with BmNPC2 proteins for immunoprecipitation. The coimmunoprecipitation samples were then analyzed by Western blotting. As shown in Fig. 4C and D, gp64 and NPC1-C proteins could be detected in the immune pellets by anti-V5 monoclonal antibody. The same immune pellets were subsequently probed with an anti-Flag antibody, and a specific band corresponding to the NPC2 in each blot was observed (Fig. 4C and D). Next, we performed mutual coimmunoprecipitation experiments, in which NPC2 protein-attached beads through mouse anti-Flag antibody were incubated with gp64 or NPC1-C proteins. NPC2’s presence in the retrieved beads was confirmed with an anti-Flag antibody (Fig. 4C and D). The band equivalent to NPC1-C and gp64 was present in the immune pellets.

### BmNPC2 enhances vesicular trafficking of viruses to BmNPC1.

**FIG 5:**
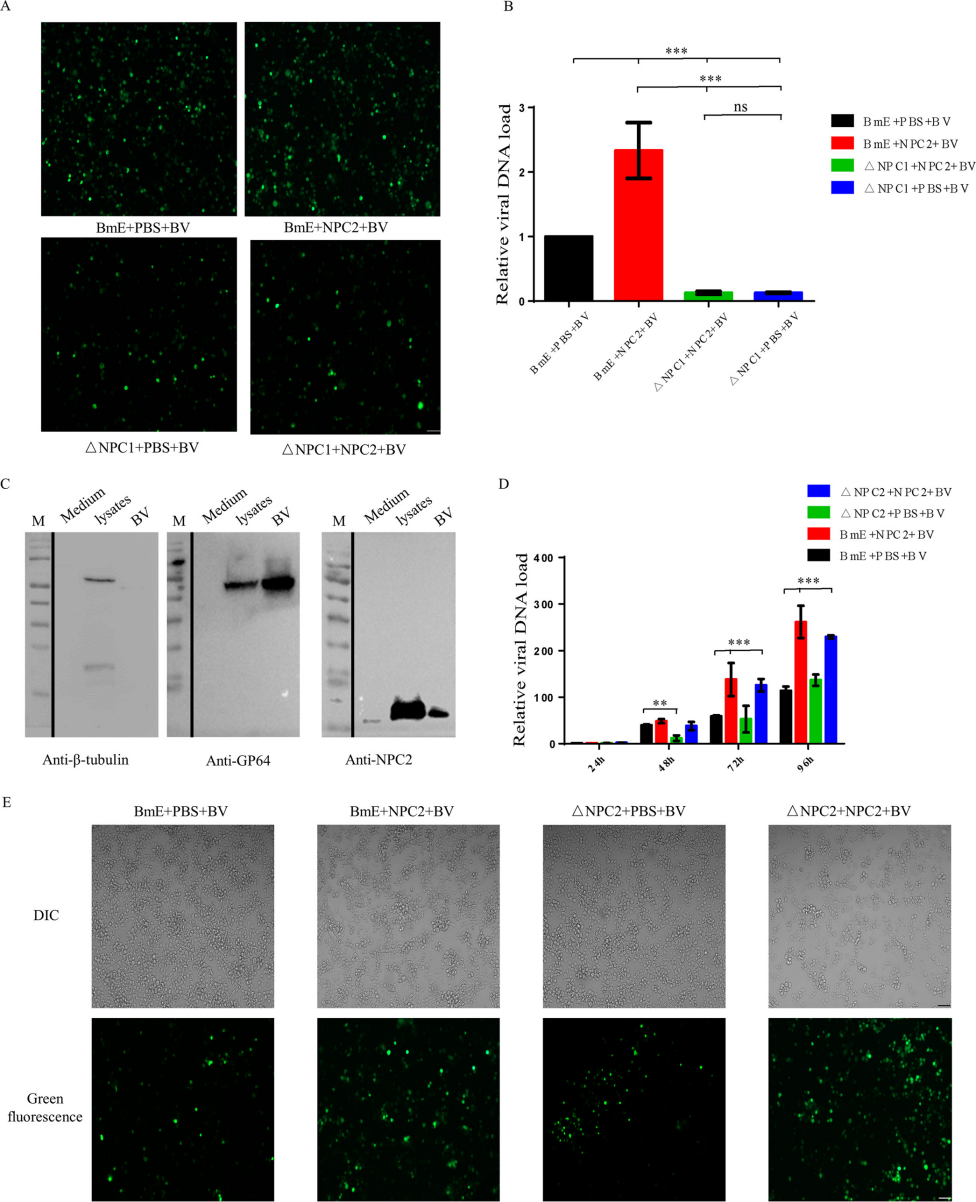
BmNPC2 enhances vesicular trafficking of viruses to BmNPC1. (A) Fluorescence microscopy of BmNPV-infected BmE and BmNPC1-null cells with or without BmNPC2 protein at 72 h. Bar, 200 μm. (B) BmE and BmNPC1-null cells with or without BmNPC2 protein and incubated with BmNPV for 72 h at 28°C. Total DNA was extracted from infected cells, and the BmNPV viral DNA level was determined by qPCR with *B. mori gadph* DNA as the endogenous control. Statistical analysis was conducted by one-way ANOVA followed by Dunn’s multiple comparison test. ns, not significant; ***, *P < *0.001. Data are means ± SD (*n* = 3). (C) Anti-BmNPC2, anti-gp64, and anti-beta-tubulin antibodies were used for Western blotting to detect cell culture medium, purified BV particles, and infected cell lysates, respectively. The virus and uninfected cell culture medium purified by the sucrose cushion were resuspended with 100 μL of PBS, and 25 μL of protein loading buffer was added. Virus-infected cells were lysed with 100 μL of radioimmunoprecipitation assay lysis buffer, and 25 μL of protein loading buffer was added. The purified virus and uninfected culture medium were loaded with the same volume, and the loading amount of the cell lysate fraction was consistent with the total protein amount of the purified virus fraction. (D) BV infects NPC2-null cells. After 72 h of infection, the progeny virus was purified. The purified BVs (1 × 10^10^ PFU) were incubated with NPC2 protein (500 μg) at 4°C for 8 h. The samples were then ultracentrifuged using a sucrose cushion to remove nonbinding NPC2 proteins, and then infected BmE cells and NPC2-null cells were incubated with BmNPV (MOI, 0.1) for 96 h at 28°C. Total DNA was extracted from infected cells, and the BmNPV viral DNA level was determined by qPCR with *B. mori gadph* DNA as endogenous control. One-way ANOVA followed by Dunn’s multiple comparison test were used for the statistical analysis. **, *P < *0.01; ***, *P < *0.001. Data are means ± SD (*n* = 3). (E) Fluorescence microscopy of BmNPV-infected BmE and BmNPC2-null cells with or without BmNPC2 protein at 96 h. Bar, 200 μm.

Next, we investigated whether NPC2 protein promotes BmNPV infection through the NPC1-NPC2 endosome cholesterol transport pathway. For this purpose, BmE cells and BmNPC1-null cells were preincubated with NPC2 proteins followed by BmNPV infection (MOI, 0.5) at 28°C for 72 h, and viral gene copy numbers were quantified by qPCR as indicated. We found that the percentage of EGFP-positive cells among BmNPC1-null cells decreased dramatically compared to the control cells at 72 h p.i. (Fig. 5A). qPCR analysis showed that viral infectivity decreased substantially in BmNPC1-null cells at 72 h p.i. (Fig. 5B). This result shows that the addition of NPC2 proteins in NPC1 knockout cell lines cannot promote virus infection, but it can be promoted in BmE cells. To investigate whether BmNPC2 was incorporated into progeny virus particles, BmE cells were infected with BmNPV at an MOI of 0.5 for 72 h or cultured without virus addition for 72 h, and then the virus-uninfected cell culture medium and virus-infected cell supernatant were collected, protein or virus was purified by the sucrose cushion method, and the cell pellets were collected at the same time. The presence of BmNPC2 protein in these fractions was detected by Western blotting. As shown in Fig. 5C, we found that BmNPC2 was present in purified virions and virus-infected cell lysates, as well as in uninfected cell culture medium, and BmNPC2 protein was significantly higher in purified virion groups than in uninfected cell culture medium. This may be due to the interaction of the viral glycoprotein gp64 with NPC2, which binds the NPC2 protein on the surface of the virion. These data suggest that NPC2 promotes the fusion efficiency of BmNPV. We next determined whether binding to NPC2 affects progeny virion infection. To test this, we first purified budding virus (BV) in NPC2-null cells. The purified BVs were incubated with NPC2 protein at 4°C for 8 h. The samples were then supercentrifuged using a sucrose cushion to remove unbound NPC2 proteins and then used to infect BmE cells and NPC2-null cells. After 96 h of infection, we found that BV-binding NPC2 protein promotes infection in BmE and NPC2-null cells (Fig. 5D and E). This indicates that progeny virions binding NPC2 protein can promote its infection, producing a cascade amplification effect. The above results indicate that NPC2 can bind to the virus and promote progeny virion infection through the NPC1-NPC2 endosome cholesterol transport pathway.

## DISCUSSION

**FIG 6:**
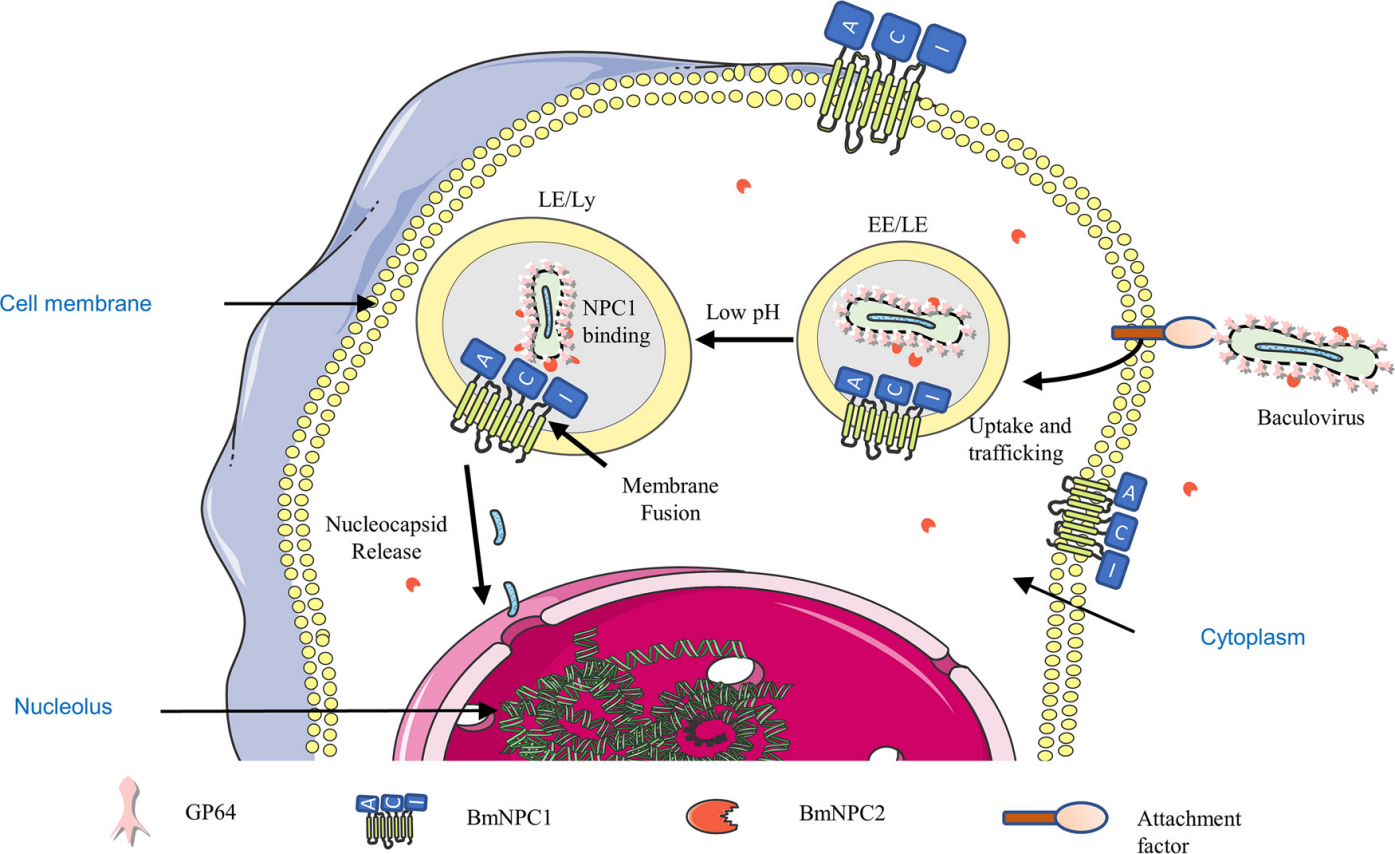
Model for the role of the NPC2-NPC1 cholesterol transport pathway in endosomes and lysosomes in baculovirus infection. The virus binds to NPC2 protein and then adheres to the cell membrane by binding to one or more cell attachment factors. They are then trafficked to LE/Ly compartments, where the structure of the gp64 protein and NPC1 protein changes, exposing the binding site under low-pH conditions. BmNPV gp64 can interact with the BmNPC1-C domain and NPC2 protein that bring about viral membrane fusion and cytoplasmic release of the viral nucleocapsid payload.

In this study, we show that after knocking out NPC1, the early stage of virus infection was greatly inhibited, mainly affecting the fusion between the viral membrane and endosomal membrane. NPC1 protein is an intracellular factor for baculovirus infection. Unlike NPC1’s housekeeping role in lysosomal cholesterol transportation, which needs all three of its major luminal loop domains, we demonstrate that NPC1’s function as a baculovirus factor requires only its second luminal loop domain (NPC1’s C domain), which engages baculovirus gp64 directly. Similarly, we knocked out NPC2 and found that the early stage of viral infection was inhibited, which affected the efficiency of viral fusion in the endosome. The NPC2 protein directly interacts with the viral envelope glycoprotein gp64 and the host NPC1 protein. The addition of NPC2 protein in the NPC1 knockout cell lines did not promote virus infection. Our findings indicate that baculovirus uses host molecules other than NPC1 to attach to the cell surface and internalize into endosomes. Based on the above results, we propose a model for the role of the cholesterol transport pathway in baculovirus entry (Fig. 6). Viral particles bind to the NPC2 protein extracellularly, attach to the cell membrane by binding to one or more cell attachment factors, and are then internalized and transported to late endosomes. In late endosomes, an acidic pH environment triggers a conformational change in gp64 to expose certain domains for better binding of the BmNPC1-C region. The gp64-NPC1 interaction may lead to viral membrane fusion and cytoplasmic release of the viral nucleocapsid.

Although BmNPC1 domain C plays a key role in viral infection, our failure to inhibit this domain alone to abolish infection implies that other domains of NPC1 may also be involved in viral infection. These sequences (domains) can better fold domain C to participate in endosomal and lysosomal delivery, either through direct contact with viral gp64 or through other molecule-assisted NPC1-dependent steps into downstream direct gp64-NPC1 interaction binding. SfNPC1 is essential for AcMNPV to infect Sf9 cells. NPC1 antagonists U18666A (0.5 to 10 μM for 24 h) could inhibit AcMNPV infection in Sf9 cells (data not shown). The sequence similarity between SfNPC1 and BmNPC1 was 64%. AcMNPV replicates in Spodoptera frugiperda cells (Sf9), but not in *B. mori* cells (BmE); conversely, BmNPV replicates in BmE cells but not Sf9 cells. In our study, the expression of BmNPC1 in Sf9 cells allowed BmNPV to enter the nucleus but failed to replicate. Two insect alphabaculoviruses, BmNPV and AcMNPV, share an ~93% amino acid sequence similarity (30) but exhibit nonoverlapping host specificity. Thus far, several genes, such as DNA helicase (31, 32) and gp64 (33), have been suggested as determinants of the BmNPV and AcMNPV host range, but the mechanism is still unclear (2). The difference in viral tropism may be related to both viruses themselves and the subtle differences between the two host cells.

Previous biochemical studies implied that the transfer of cholesterol from NPC2 to NPC1 is a critical part of the efflux mechanism. NPC1 domain C could bind directly to cholesterol-loaded NPC2 *ex vivo* at acid pH but not at neutral pH (34). These results support a model in which docking of NPC2 to NPC1 domain C facilitates cholesterol transfer from NPC2 to cholesterol-binding domain A of NPC1. In contrast to the NPC1 interaction with NPC2, NPC2 protein and NPC1-C domain protein interact with viral envelope glycoprotein at a low level of cholesterol and neutral pH. Meanwhile, it remains unknown whether NPC2 protein enhances the binding affinity of NPC1 to gp64 protein and whether low pH is the trigger of NPC1-mediated baculovirus envelope fusion. Under low-pH conditions, the conformations of NPC1 and gp64 proteins change, which in turn induces the fusion of the viral envelope and endosomal membrane, and then viral nucleocapsid is released into the cytoplasm.

It has been demonstrated recently that filoviruses, including Marburg viruses and Ebola, utilize NPC1 as intracellular receptors for entry (35). Interestingly, apart from EBOV (family *Filoviridae*), hepatitis C virus (family *Flaviviridae*) entry also requires the cholesterol trafficking receptor Niemann-Pick C1-like 1 (36). In our study, we not only presented the evidence that baculovirus relies on the host factor NPC1 to enter into insect cells but also demonstrated that NPC2, another important protein in the cholesterol transport pathway, could interact with the viral envelope glycoprotein gp64 to promote BmNPV infection through the intracellular cholesterol transport pathway. Previous research results did not provide direct evidence that NPC2 protein is related to virus infection. Here, we found that NPC2 protein interacts with viral envelope glycoprotein gp64 to promote virus infection through the intracellular cholesterol transport pathway. We believe that our study on the BmNPV entry mechanism will further facilitate the application of baculovirus systems in eukaryotic gene delivery. Not only can the cholesterol transport pathway NPC1 protein be used by a variety of enveloped viruses, but also the NPC2 protein can be used by viruses to infect host cells. This will provide new insights into the study of enveloped virus infection mechanisms.

## MATERIALS AND METHODS

### Cells and virus production and purification.

Bombyx mori cell line BmE-SWU cells were maintained at 28°C in Grace’s medium (Thermo Fisher Scientific, MA, USA) supplemented with 10% (vol/vol) fetal bovine serum (FBS; Thermo Fisher Scientific, MA, USA) and 1%b(vol/vol) penicillin-streptomycin (37). The Sf9 cell line (Thermo Fisher Scientific, MA, USA) was cultured at 28°C in SF900II insect medium (Thermo Fisher Scientific, MA, USA) using standard techniques. Recombinant virus constructs have been described previously (38). In brief, the *egfp* gene was inserted under the p10 promoter to generate recombinant plasmid pFastBac-egfp. BmNPV bacmid (BmBac-EGFP) was constructed using pFastBac-egfp according to the Bac-to-Bac system manual of Invitrogen (39). The recombinant bacmid was transfected into BmE cells for virus preparation, and virus titer was determined in an endpoint dilution assay. The recombinant virus AcBac-EGFP was generated by transforming AcDH10Bac Escherichia coli (Invitrogen, USA) and transfecting the indicated bacmids into Sf9 cells according to the manufacturer’s instructions (Bac-to-Bac baculovirus expression system; Invitrogen, USA), and viral titers were determined in the endpoint dilution assay. To produce VP39-EGFP-labeled baculoviruses (VP39-EGFP), the *vp*39 gene was amplified and then fused to the *egfp* gene via PCR amplification using previously described methods (40). The amplified DNA was then cloned into the pFastBac Dual construct, and the recombinant baculovirus VP39-EGFP was generated with the above-mentioned method. The BVs were purified, first by pelleting through an ultracentrifugation at 200,000 × *g* for 2 h at 4°C (Sorvall WX80+; Thermo Fisher Scientific, USA). Next, we concentrated the supernatant through a 25% sucrose cushion and then resuspended it in 100 μL PBS (41). Culture medium of uninfected BmE cells was also purified by this method as a negative control for the experiment.

### Cloning, protein expression, purification, and antibody production.

The sequence of BmNPC1 extracellular loop C (residues 408 to 645) was amplified with primer pairs BmNPC1-C and then cloned into pET32a plasmid for protein expression in E. coli. The recombinant BmNPC1-C proteins with His tag were purified with a HisTrap HP 5-mL column (GE Healthcare, USA) according to the manufacturer’s instructions. To generate an NPC2 expression recombinant AcMNPV bacmid, the full-length NPC2 coding domain sequence with its native signal peptide fused with a 6× His tag and Flag tag was amplified and cloned into donor plasmid, under the control of pH promoter of pFastBac Dual (Thermo Fisher Scientific, MA, USA). The resulting plasmids were subsequently electroporated into E. coli DH10Bac cells harboring Ac-Bacmid and helper plasmids to generate recombinant bacmids. Sf9 cells (3 × 10^5^ cells per 35-mm well) were transfected with 2.0 μg of the appropriate bacmid DNA (AcBac-NPC2), and expression was confirmed by Western blotting. The recombinant BmNPC2 protein was purified as described elsewhere (28). The purified recombinant protein BmNPC2 was subsequently used as an immunogen to immunize 7-week-old BALB/c mice with Freund’s complete adjuvant (Sigma-Aldrich, USA). The anti-BmNPC2 mouse serum and control sera from naive mice were collected and stored at −20°C until use.

### Construction and verification of NPC2-knockout (ΔNPC2) BmE cells.

The CRISPR/Cas9 genomic editing tool was used to generate the stable BmNPC2-knockout BmE cell line. The Cas9 expression vector (named pSL1180-IE1-Cas9) and single guide RNA (sgRNA) expression vector (named PUC57-U6-gRNA) were constructed as previously described (11) (see Fig. S1A in the supplemental material). The primer sequences generating the guide RNA (gRNA) targeting the BmNPC2 gene were designed based on the CRISPR website (http://crispr.dbcls.jp/). All candidate sgRNA target sequences bear the GN19NGG sequence. To construct the ΔNPC2 cell lines, we followed a protocol established by Li et al. (11).

### Filipin staining.

Extemporaneous preparation of the filipin staining solution was described previously (42). BmE cells and ΔNPC2 cells were seeded on coverglasses in 12-well plates (Corning, USA) for 12 h at 28°C. The cells were rinsed with PBS twice (5 min each time), followed by the incubation with 0.5 mL/well filipin (40 μM/mL) staining solution in the dark for 45 min. Next, the cells were rinsed twice for 3 to 5 min with PBS and visualized with an Olympus confocal microscope.

### Immunofluorescence microscopy.

Cells were seeded onto coverslips and infected with VP39-EGFP (MOI of 80 50% tissue culture infective dose [TCID_50_] units/cell). Cells were then fixed at 0, 30 min, 1 h, and 2 h (BmE cells, ΔNPC1 cells, and ΔNPC2 cells). Immunofluorescence microscopy was performed using anti-EGFP mouse monoclonal antibody (Roche Diagnostics, Germany) and Alexa Fluor 488–goat anti-mouse antibody (Sigma-Aldrich, USA). Cells were counterstained with DAPI to stain the nuclei and imaged with an Olympus confocal microscope.

### Fluorescence labeling of BmNPV.

Viruses were propagated, purified, and labeled with a self-quenching DiO dye at a concentration of 200 μM as previously described (43). Labeled viruses were passed through a Zeba spin desalting column (Thermo Fisher Scientific, USA) and a 0.45-μm filter to remove excess dye and aggregates, respectively, before being aliquoted and stored at –80°C until imaging.

### Live-cell imaging.

Live-cell imaging of virus binding and internalization was performed with an Olympus confocal microscope. Cells were transfected with plasmids expressing BmNPC1 for 48 h before incubating with VP39-EGFP at an MOI of 80 TCID_50_ on ice for 1 h followed by an extensive wash with cold PBS. The cells were then incubated with cold culture medium and visualized live with a confocal microscope. To visualize virus entry, the culture medium was replaced with prewarmed (28°C) culture medium to trigger virus internalization, and the images were acquired.

### Virus binding.

Cells were precooled at 4°C for 30 min and were then inoculated with BmNPV (MOI, 80) at 4°C for 60 min. Cells were then collected and washed with cold PBS 3 times to remove the unbound viruses, and the cell-bound viral genomes were quantified by qPCR with primers against the viral gene *gp41* as previously reported (11).

### Western blotting.

Western blotting was performed as previously described (11). Cells were washed twice with PBS and lysed with protein lysis buffer (Beyotime, Shanghai, China) containing protease inhibitor cocktail (MCE, USA) and 2% Triton X-100. After 30 min on ice, lysates were treated with 20% loading buffer (Beyotime) for 10 min at 100°C. Proteins were resolved by SDS-PAGE and analyzed by immunoblotting using anti-V5 antibody (1:2,000; Abcam, Cambridge, USA), anti-beta-tubulin antibody (1:1,000; abm, Canada), anti-Flag antibody (1:2,000; Sigma, Burghausen, Germany), anti-gp64 antibody (1:2,000; Abcam, Cambridge, USA), and anti-NPC2 antibody (1:1,000).

### *In vitro* expression of BmNPC2, BmNPC1-C, and gp64 proteins and coimmunoprecipitation.

Coimmunoprecipitation was performed based on a protocol developed by Dong et al. (10). Briefly, we overexpressed NPC2 and gp64 simultaneously in BmE cells. At 72 h posttransfection, cells were washed twice with PBS and lysed with protein lysis buffer (Beyotime) containing protease inhibitor cocktail (MCE, USA) and 2% Triton X-100. After 30 min on ice, lysates were centrifuged at 14,000 × *g* for 30 min, and the supernatants were collected in a 1.5-mL tube. A 50-μL aliquot of protein G-agarose beads (Bio-Rad, USA) was incubated with 1 μg anti-Flag or anti-V5 antibody diluted in 500 μL PBS with Tween 20 for 30 min at room temperature, then placed on the magnet to remove the supernatant and to bear the cell lysate supernatants for 8 h at 4°C, and then placed on the magnet to remove the supernatant. The beads were then washed 3 times in PBS. Then, 40 μL of lysis buffer and 10 μL of loading buffer were added and heated for 10 min in a water bath at 100°C. The eluted proteins were detected with anti-V5 antibody and anti-Flag antibody, respectively. The same method was used to detect the interaction between NPC2 and NPC1-C, except for the low-pH conditions used for incubation.

### Yeast two-hybrid assay.

The yeast two-hybrid assay was used to confirm the interaction between NPC2, NPC1-C (residues 408 to 645), and gp64 (residues 18 to 499) *in vitro* according to a previously described method (44). The bait and prey construct pairs pGBKT7-NPC2/pGADT7-NPC1-C, pGBKT7-NPC2/pGADT7-gp64, and pGBKT7-NPC1-C/pGADT7-NPC2, pGBKT7-GP64/pGADT7-NPC2 were transformed simultaneously into competent yeast cells to examine the protein interaction. All primers used are listed in Table S1 in the supplemental material.

### Protein blocking assay.

Wild-type cells were seeded at 3 × 10^5^ cells/well in 12-well culture plates 1 day prior to infection. BmNPV (MOI, 0.3) were incubated with purified, soluble domain C of BmNPC1 or Trx tag protein of pET-32a vector at 4°C overnight and then were added to the cells. At 72 h p.i., infected cells were imaged with a fluorescence microscope, and then the cells were harvested for measuring viral DNA load by qPCR as described previously (11).

### CCK8 assay.

Survival curves of BmE, NPC2 knockout cells were assessed in a cell counting kit 8 (CCK8) assay (Yeasen, Shanghai, China). Briefly, cells were seeded in 96-well plates at a density of 1 × 10^4^ cells per well, allowed to adhere for 24 h at 28°C, and then incubated for 24, 48, 72, and 96 h. The samples were assayed in at least three independent experiments, and the mean values for each experiment were calculated.

### Statistical analyses.

Graphical representation and statistical analyses were performed using Prism6 software (GraphPad Software, USA). All data presented are representative of a minimum of three independent experiments. Group results are expressed as mean values ± standard deviations (SD). Data between two groups were compared using paired two-tailed *t* tests. Data among multiple groups were compared using a one-way analysis of variance (ANOVA) followed by Dunn’s multiple comparison test.
