# Supplementary material for: The NPC Families Mediate BmNPV Entry
Source: Microbiol Spectr. 2022 Jul 5;10(4):e00917-22. doi: 10.1128/spectrum.00917-22 (PMC9430594; doi:10.1128/spectrum.00917-22)
Supplement: Supplemental file 1 — Fig. S1 and Table S1. Download spectrum.00917-22-s0001.pdf, PDF file, 0.3 MB [file spectrum.00917-22-s0001.pdf]

Supplemental Materials

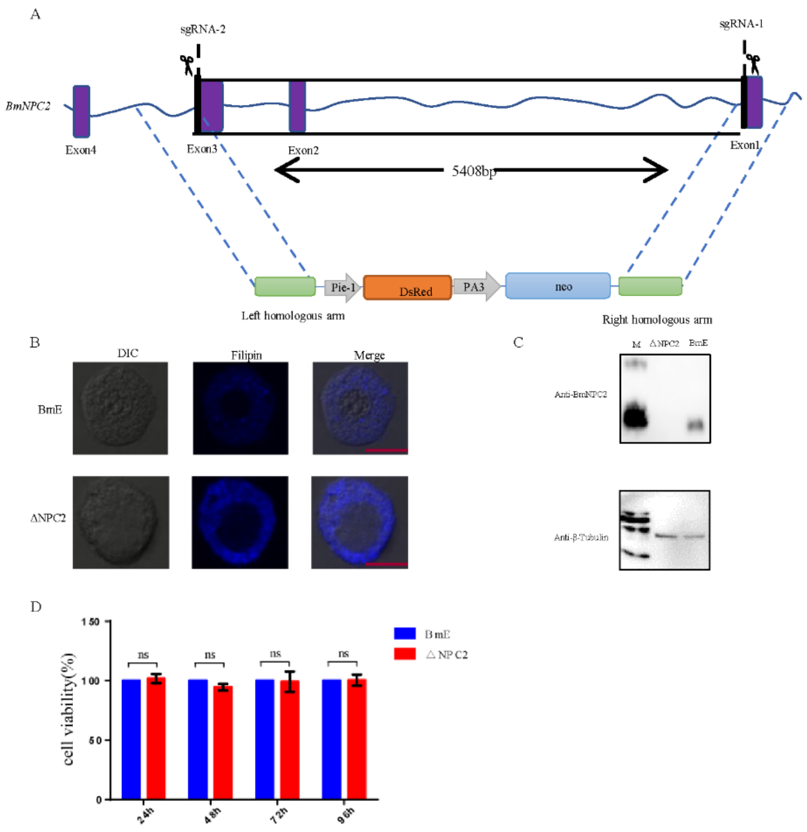

Fig. S1 Generation of BmNPC2 knockout BmE cell using CRISPR/CAS9 system.

(A) Schematic structure of Cas9 and gRNA expression vector for BmE cell. Cas9 was driven with *ie-1* promoter and ended with SV40 terminator. Chimeric gRNA contained U6 promoter, chimeric site, gRNA1 scaffold, and terminal sequence (TTTTTT). Schematic for homologous recombination (HR) mediated by Cas9. Homologous arms (light green) were PCR amplified from BmE cells genomic DNA with specific primers (Supplementary Table S1). The Left-HR, DsRed expression cassettes, *neo* expression cassettes and Right-HR were sequentially cloned into the pEASY vector to generate donor plasmid. (B) The cholesterol staining of BmE cells and ΔNPC2 cells with filipin. BmE cells and ΔNPC2 cells were stained by filipin (blue). Bar, 5 μm. (C) Verification

of BmNPC2 knocked out BmE cells by Western Blotting. Proteins were resolved by 15% SDS-PAGE and analyzed by immunoblotting using anti-Beta-Tubulin antibody (1:1000, abm, Canada), anti-NPC2 antibody (1:1000) respectively. (D) CCK8 assay was used to detect the viability of NPC2 knocked out cell. Statistical analysis was conducted by paired two-tailed t-tests. ns, no significance.

Table S1 Sequences of primers used in this study.

Primer name	Primer sequence	Amplicon size (bp)
Recombinant BmNPV primers		
pFastBacHTB-EGFP-F	5' CGGAATTCGTGAGCAAGGGCGAGGAG 3'	731
pFastBacHTB-EGFP-R	5' CCGCTCGAGCTTGTACAGCTCGTCCAT 3'	
CRISPR/Cas9 system primers		
sgNPC2-1-F	5' AAGTGGCTATGCCGGGAAGGTTAG 3'	24
sgNPC2-1-R	5' AAACCTAACCTTCCCGGCATAGCC 3'	
sgNPC2-4-F	5' AAGTGCATTGTAGAGAGCATCAAA 3'	24
sgNPC2-4-R	5' AAACTTTGATGCTCTCTACAATGC 3'	
Left-HR-F	5' GGGCCCTTTATGCGGTATGTTAGC 3'	1050
Left-HR-R	5' CTCGAGAAGTTGAATTTCGATTGG 3'	
Right-HR-F	5' CCCGGGCACGTCAGTGGCCTTATGTTAATCT 3'	1463
Right-HR-R	5' CCGCGGGAATGCATTAATGGATCCACATGTT 3'	
*ie*1-DsRed-sv40-F	5' CTCGAGATCGATGTCTTTGTGATG 3'	1535
*ie*1-DsRed-sv40-R	5' GAATTCTAAGATACATTGATGAGT 3'	
A3-*neo*-sv40-F	5' GAATTCCTCAAGCTTGATGCGCGTTA 3'	1888
A3-*neo*-sv40-R	5' CCCGGGTAAGATACATTGATGAGT 3'	
Knock out detect primers		
KO-detect-left arm-F	5' GGGGTAAGGTACTGAAATCCCG 3'	3097
KO-detect-left arm-R	5'CAGGAACAGGTGGTGGCGGCCCT 3'	
KO-detect-right arm-F	5’ATGGTGCGCTCCTCCAAGAACGTC 3’	2036
KO-detect-right arm-R	5' CCGCTGCCTACCGTTAAGTACTCTCCA3'	
qPCR primers		
GP41-q-F	5'AGCGGTTTGAATCGGAGGAGAC 3'	283
GP41-q-R	5'TTGAACGGTAACGGAATGGGAA 3'	
BmGAPHG-q-F	5'CATTCCGCGTCCCTGTTGCTAAT 3'	103
BmGAPHG-q-R	5'GCTGCCTCCTTGACCTTTTGC 3'	
Cell expression protein primers		

F-NPC2-TY	ATCTGTTCGAATTTAAAGCTTGGTACCATGGCTCTCTACTCTTCGCTG	668
R-NPC2-TY	TCGAACCGCGGGCCCTCTAGACTCGAGAACAAGCCTGACATTCGTTCT	722
R-FLAG	CGCGGGCCCTCTAGACTCGAGTTACTTATCGTCGTCATCCTTGTAATCCGATCCGCCGC	
F-NPC1-C-TY	TCGAATTTAAAGCTTGGTACCATGACCGACCCCGTGGAGCTG	830
R-NPC1-C-TY	CCGCCGCCCCCAGAGCCACCTCCGCCAGAGCCGCCTCCGCCGGACTCGCGGTCCAGCTC	
F-GP64-TY	ATCTGTTCGAATTTAAAGCTTGGTACCATGCTACTAGTAAATCAGTCA	1514
R-GP64-TY	TTACCTTCGAACCGCGGGCCCTCTAGACGAAGTCAATTTGGCGGC	
Yeast two-hybrid system primers		
GP64-yeast-F	GGAATTCCATATGATGCTACTAGTAAATCAG	1519
GP64-yeast-R	TCCCCCGGGCGAAGTCAATTTGGCGGC	
NPC1-C -yeast–F	GGAATTCCATATGACCGACCCCGTGGAGCTG	724
NPC1-C -yeast–R	TCCCCCGGGGGACTCGCGGTCCAGCTC	
NPC2-yeast-F	GGGAATTCCATATGATGGCTCTCTACTCTTCGCTG	462
NPC2-YEAST-R	CGCGGATCCAACAAGCCTGACATTCGT	
